# Entropy, Inhibition and Memory in Balanced Spiking Reservoirs

**DOI:** 10.3390/e28070784

**Published:** 2026-07-10

**Authors:** Luigi Rosati, Nicola Toschi, Andrea Duggento

**Affiliations:** 1 Department of Biomedicine and Prevention, University of Rome Tor Vergata, 00133 Roma, Italy; nicola.toschi@uniroma2.eu (N.T.); andrea.duggento@uniroma2.eu (A.D.); 2 A. A. Martinos Center for Biomedical Imaging, Harvard Medical School, Charlestown, MA 02129, USA

**Keywords:** spiking neural networks, reservoir computing, RNN, Brunel network

## Abstract

Recurrent neural networks are studied along two largely parallel tracks: as machine-learning models evaluated by task performance and as computational-neuroscience models of cortical circuits evaluated by dynamical realism. Reservoir computing offers a meeting point, yet the link between dynamical regime and computational performance has not been systematically mapped in biologically constrained spiking architectures. We treat the Brunel balanced excitatory–inhibitory network as a reservoir and characterize separation capacity (kernel quality) and transient memory (corrected linear memory capacity, validated by non-parametric mutual information) across the full phase diagram. The analysis uses a four-state Markov source whose Shannon entropy rate is set in closed form by a single parameter at fixed marginal entropy. Both capabilities increase monotonically with the inhibitory ratio *g*, remaining jointly highest in the asynchronous irregular regime, with diminishing increments consistent with eventual saturation; the synchronous irregular regime, despite a network timescale three orders of magnitude longer, supports neither. Memory further requires sparse input coupling: dense coupling collapses the driven timescale and erases memory in every regime. Inhibitory balance thus emerges as a unified architectural control parameter, providing a quantitative design criterion for cortical-circuit modeling and reservoir computing applications.

## 1. Introduction

Recurrent neural networks (RNNs) lie at the intersection of machine learning and computational neuroscience, yet the two communities have developed largely parallel traditions and rarely share a common evaluation framework, despite growing recognition that progress in either field benefits from explicit cross-translation between cellular-level mechanisms and computational architectures [1]. In machine learning, RNNs are trained end-to-end by gradient-based optimization and evaluated by task performance, with internal dynamics treated as an implementation detail [2,3]. In computational neuroscience, recurrent networks are studied as models of cortical circuits, with detailed analyses of spontaneous activity, regime structure, and population geometry, but the link to specific computational tasks is often secondary [4,5,6,7,8]. The two literatures typically use different network classes (rate units versus spiking neurons), different observables (loss curves versus firing-rate distributions), and different notions of success (generalization versus biological plausibility). Consequently, the relationship between the dynamical properties of a recurrent network and its computational capabilities is rarely articulated in terms that both sides can use. The aim of the present work is to construct one such common framework: to take a network model that is canonical in computational neuroscience, evaluate it with the metrics that are canonical in reservoir computing, and report the results in a way that is interpretable from either side.

Reservoir computing (RC) was chosen because it offers a tractable framework at this intersection [9,10,11]. The recurrent network, or reservoir, is fixed and untrained, while only a linear readout adapts to the task. Computational performance is therefore determined directly by the dynamical properties of the reservoir, and the link between network dynamics and task capability is exposed without the confound of training-induced representational changes. A prominent trend within the RC literature is to use biologically constrained spiking networks as reservoirs, in the spirit of the original liquid state machine proposal [10,12]: such networks respect Dale’s law, separate excitatory and inhibitory populations, and use sparse random connectivity with cell-type-specific connection probabilities and realistic synaptic time constants. The motivation is that they may capture properties of real cortical circuits and yield more interpretable models of neural computation. In the fully spiking-RC literature, however, the dynamical regime of the reservoir is rarely characterized or controlled: studies focus on matching anatomical statistics rather than on selecting a specific dynamical phase, and the connection between regime and performance is left implicit.

The conceptual gap has a clear cause: spontaneous dynamics and input-driven computational properties have largely been studied with different goals and metrics. Computational neuroscience has emphasized how recurrent parameters determine activity regimes and population statistics, while RC has measured how networks transform input sequences (separation, memory, scaling with size). The bridge between the regime a balanced spiking network occupies and its computational performance as a reservoir has not been established systematically. In its absence, practitioners rely on heuristics such as the edge-of-chaos criterion [13,14,15], which states that networks near the order–chaos boundary maximize representational diversity and the lifetime of input-evoked transients, while useful for rate networks and binary threshold gates, this heuristic has not been validated empirically across a full spiking phase diagram.

We close this gap by adopting the Brunel (2000) balanced excitatory–inhibitory spiking network as the reservoir, precisely because its phase diagram is analytically characterized [5], exposing four dynamical regimes (asynchronous irregular, synchronous regular, synchronous irregular, fast oscillations) within a single biologically motivated architecture. We use kernel quality [14] and corrected linear memory capacity [9,16,17] as the established RC metrics, complemented by a non-parametric mutual information estimator [18] as a model-free validation; the experimental design is detailed in Section 2.

Two recent studies on the dynamics–computation link in spiking reservoirs motivate this protocol by contrast. Kanamaru et al. [19] report maximal memory capacity near the edge of chaos, but in a multi-module cortical-layer assembly analyzed through ensemble Fokker–Planck equations and a single E-I balance parameter, whereas ours is the monolithic Brunel LIF network analyzed at the level of individual spike trains, sweeping the full (g,η) phase diagram and measuring separation alongside memory within one protocol. Woo et al. [20] characterize liquid state machines through neuronal avalanches and functional clustering, linking scale-invariant dynamics to classification performance; their analysis is task-driven and centered on self-organized criticality, while ours is task-agnostic and grounded in the analytic phase structure of a canonical balanced model. Both support the regime–performance link we examine here while leaving the joint mapping from dynamical phase to separation and memory unaddressed.

To separate intrinsic properties of the network from properties of the input, we drive the reservoir with a symmetric Markov source whose Shannon entropy rate is controlled analytically by a single parameter at a fixed marginal distribution. This explicit decoupling of network state and input statistics is, to our knowledge, not standard in the spiking-RC literature, where the influence of input properties on reservoir computation has been examined only in fragments: the input-distribution dependence of memory capacity in echo state networks [21], the generalization of memory capacity to autocorrelated stationary signals [22], the suppression of chaos by deterministic stimuli in rate networks [23], and the role of input correlations in balanced rate networks [24]. None of these studies sweeps a continuous, analytically tractable input-entropy axis on a spiking phase diagram at fixed marginal.

Each element of the design pairs a biophysical control parameter with a machine-learning observable, and the resulting map is intended as a concrete instance of how cortical-circuit models and recurrent sequence computation can be analyzed in shared, quantitative terms. We do not propose a new network architecture; instead, we offer a unifying read-out of an established model under metrics that have so far been applied to it only in scattered form, with the aim that the same protocol be transferable to other biophysical models of recurrent computation. The paper is organized as follows: Section 2 describes the network model, input source, coupling protocol, and metrics; Section 3 presents the spontaneous and input-driven characterizations; the Section 4 and Section 5 then interpret the findings and outline open questions, with calibration sweeps and supporting analyses in the Appendix A.

## 2. Materials and Methods

The study consists of two coordinated experimental phases on the same balanced spiking architecture. A spontaneous characterization phase sweeps the Brunel (g,η) parameter space across multiple network sizes to identify the smallest size at which all four dynamical regimes coexist stably. An input-driven phase, conducted at the selected operating size, projects a four-state Markov sequence with controlled autocorrelation into the network and quantifies separation, transient memory, and mutual information across the same (g,η) grid. The remainder of this section describes the network model (Section 2.1), the input source (Section 2.2), the input-to-reservoir coupling (Section 2.3), and the computational metrics (Section 2.4). Simulation and analysis code are available at https://github.com/luigirosati/entropy-reservoir (accessed on 26 May 2026).

### 2.1. Reservoir Network

The reservoir is a balanced excitatory–inhibitory (E/I) spiking network following Brunel [5]: N=5000 leaky integrate-and-fire (LIF) neurons with alpha-function synapses on a sparse random recurrent graph. A fraction $N_{E}=0.8N$ are excitatory and $N_{I}=0.2N$ inhibitory, in agreement with Dale’s law. Each neuron receives $C_{E}=c\cdot N_{E}$ excitatory and $C_{I}=c\cdot N_{I}$ inhibitory recurrent inputs drawn independently with connection probability c=0.1. The subthreshold membrane potential *V* evolves as

(1) $$\tau_{m}\frac{dV}{dt}=-V+R_{m}I\left(t\right),$$

with $\tau_{m}=20\;\mathrm{ms}$ and I(t) the total synaptic current. A spike is emitted when *V* reaches $V_{\mathrm{th}}=20\;\mathrm{mV}$ (relative to rest); the membrane is then clamped at $V_{\mathrm{reset}}=10\;\mathrm{mV}$ for an absolute refractory period $t_{\mathrm{ref}}=2\;\mathrm{ms}$. Synaptic currents are alpha functions with time constant $\tau_{\mathrm{syn}}=0.5\;\mathrm{ms}$. The recurrent excitatory weight is $J_{E}=0.1\;\mathrm{mV}$, and inhibitory weights are $J_{I}=-gJ_{E}$, with *g* the inhibitory ratio that sets the dynamical regime.

Each neuron also receives independent external Poisson spike trains at rate $\nu_{\mathrm{ext}}$ through $C_{E}$ excitatory synapses of weight $J_{E}$. The drive is parameterized by $\eta =\nu_{\mathrm{ext}}/\nu_{\mathrm{thr}}$, where $\nu_{\mathrm{thr}}=V_{\mathrm{th}}/\left(J_{E}C_{E}\tau_{m}\right)$ is the single-neuron firing threshold rate. The pair (g,η) thus determines the operating point in the Brunel phase diagram. All simulations were performed with NEST [25] using the iaf_psc_alpha neuron model.

The choice N=5000 comes from the multi-scale spontaneous characterization: it is the smallest size at which all four Brunel regimes coexist with stable statistics, since the SI and fast-oscillation phases require large-*N* collective synchrony (Appendix A.2). All network parameters are listed in Table 1.

### 2.2. Input Signal Generation

We adopt a discrete-time symbolic source designed to be the simplest prototype of a spike-rate-coded signal for delivery to a spiking reservoir. At each time step *k*, exactly one of $N_{\mathrm{in}}=4$ channels is active for a fixed duration $T_{\mathrm{bit}}$, and during that window, the active channel is conveyed as a Poisson spike train at rate $\nu_{\mathrm{ON}}$ to its target neurons (Section 2.3). This construction is deliberately minimal: it places the entire temporal structure of the input in the symbol sequence $\left\{S_{k}\right\}$, while leaving its biological interpretation (one channel as a one-hot rate code, the spike train as the natural encoding of that rate at the synapse) transparent. The window $T_{\mathrm{bit}}$ thereby acquires a precise dual meaning, both as the symbol duration of the source and as the integration window over which a downstream rate code would need to be read out. Selecting it is therefore a necessary step for any rate-to-spike encoding scheme, and we examine its effect explicitly in Appendix A.9.

The symbol sequence ${\left\{S_{k}\right\}}_{k=1}^{N_{w}}$ is generated by a homogeneous Markov chain with symmetric transitions

(2) $$P\left(S_{k}=j|S_{k-1}=i\right)=\left\{\begin{array}{cc} \varepsilon , & j=i,\\ \frac{1-\varepsilon }{N_{\mathrm{in}}-1}, & j\neq i, \end{array}\right.$$

where ε∈[0,1] is the stay probability. The stationary distribution is uniform for every ε, so the marginal symbol entropy is fixed at $H\left(S\right)=\log_{2}N_{\mathrm{in}}=2\;\mathrm{bits}$ (used as the normalization for mutual information in Section 2.4.3), while the conditional entropy is varied through ε alone. The Shannon entropy rate of the source is then

(3) $$H\left(\varepsilon \right)=-\varepsilon \log_{2}\varepsilon -\left(1-\varepsilon \right)\log_{2}\;\left(\frac{1-\varepsilon }{N_{\mathrm{in}}-1}\right),$$

ranging from $H\left(0.25\right)=\log_{2}N_{\mathrm{in}}=2\;\mathrm{bits}/\mathrm{symbol}$ at maximum entropy (ε=0.25, i.i.d.) down to H→0 as ε→1 (Figure A2, Appendix A.3). ε thus parameterizes a one-dimensional path through information space along which the marginal distribution is held fixed by construction, isolating the dependence of reservoir performance on the temporal redundancy of the input. This explicit, closed-form control over H(ε), together with the lag-τ autocorrelation $\lambda {\left(\varepsilon \right)}^{\tau }$ derived in Appendix A.3, is the property that makes the source suitable for an information-theoretic characterization of the network response.

### 2.3. Input Coupling

Each of the $N_{\mathrm{in}}$ channels drives a dedicated subset of reservoir neurons of size $n_{\mathrm{frac}}\cdot N_{E}$ excitatory and $n_{\mathrm{frac}}\cdot N_{I}$ inhibitory targets, with $n_{\mathrm{frac}}\in \left(0,1\right]$ controlling the input coupling density. When channel *j* is active, its target neurons receive additional Poisson spike trains at rate $\nu_{\mathrm{ON}}=100\;\mathrm{Hz}$ through dedicated excitatory synapses of weight $j_{\mathrm{in}}=J_{E}$; when inactive, the corresponding generators are silent ($\nu_{\mathrm{OFF}}=0$). Synaptic targets are drawn independently per random seed, with overlap between channels allowed.

The values $n_{\mathrm{frac}}=0.10$ and $j_{\mathrm{in}}=J_{E}$ were selected by systematic calibration sweeps reported in Section 3 and detailed in Appendix A.7 and Appendix A.8. All simulation parameters are listed in Table A1 (Appendix A.4).

### 2.4. Computational Metrics

Three metrics quantify the reservoir response to the input: separation capacity, transient memory, and mutual information with past inputs. All three are computed from the same *K*-dimensional principal component analysis (PCA) projection of the population spike-count matrix, with K=20 chosen by a dimension sweep (Appendix A.6).

#### 2.4.1. Separation Capacity

We computed the kernel quality (KQ) introduced by Legenstein and Maass [14],

(4) $$\mathrm{KQ}=\frac{E\;\left[∥X_{k}-X_{k^{\prime }}{∥}^{2}|S_{k}\neq S_{k^{\prime }}\right]}{E\;\left[∥X_{k}-X_{k^{\prime }}{∥}^{2}|S_{k}=S_{k^{\prime }}\right]},$$

the ratio of the expected squared distance between reservoir states ($X_{k}\in R^{K}$, the PCA-projected population spike-count vector at window *k*) driven by different symbols to the expected distance between states driven by the same symbol. The natural null is KQ=1, which holds whenever the reservoir state is independent of the input symbol; values above 1 indicate that distinct symbols drive the network to states more separated than the within-symbol fluctuation level. The original formulation by Legenstein and Maass [14] evaluates KQ on the full $N_{E}$-dimensional state. Our PCA projection retains the top K=20 components, and dimensions beyond K=20 contribute more within-class noise than between-class signal (memory capacity saturates at K=20, Appendix A.6). Including them would allow the numerator and denominator to grow similarly, pushing KQ toward 1. The same value of *K* is used for KQ, which is less sensitive to *K* than $C_{\mathrm{mem}}$ since class-discriminative variance concentrates in the leading components. The KQ values reported here are thus an upper bound on the full-state value under our sparse coupling.

We also computed the participation ratio (PR) of the population response [26,27],

$$\mathrm{PR}=\frac{{\left(\sum_{i}\mu_{i}\right)}^{2}}{\sum_{i}\mu_{i}^{2}},$$

where $\mu_{i}>0$ denote the positive eigenvalues of the population spike-count covariance matrix, computed efficiently via the $\min\left(N_{w},N_{E}\right)\times \min\left(N_{w},N_{E}\right)$ Gram matrix when the number of analysis windows $N_{w}$ is smaller than the number of neurons $N_{E}$ (the non-zero eigenvalues of the two representations are identical). Neurons with zero spike-count variance across windows, which are mostly silent neurons, are excluded before computing the covariance. The PR equals 1 when a single eigenvalue dominates (activity confined to one collective mode) and equals the number of active neurons when all eigenvalues are equal (uniformly high-dimensional activity). It thus quantifies the effective number of independent directions occupied by the population state. We compute it under spontaneous activity (${\mathrm{PR}}_{\mathrm{spont}}$) and under input drive (${\mathrm{PR}}_{\mathrm{driven}}$) using the corresponding spike-count matrix in each case.

#### 2.4.2. Transient Memory Capacity

Linear memory capacity [9] is estimated by regressing the reservoir state at window *k* onto the input symbol τ steps earlier:

(5) $$R^{2}\left(\tau \right)=\max_{W}\;\mathrm{CV}\;\left[{\mathrm{corr}}^{2}\;\left(WX_{k},\;S_{k-\tau }\right)\right],$$

where $X_{k}\in R^{K}$ is the PCA-projected state, $S_{k-\tau }$ is the one-hot encoding of the symbol τ steps in the past, and the optimization is over linear readouts *W*, with CV[·] denoting cross-validated evaluation. The total corrected linear memory capacity is

(6) $$C_{\mathrm{mem}}^{\mathrm{corr}}=\sum_{\tau \geq 1}\max\;\left(0,\;R^{2}\left(\tau \right)-r_{0}\;\lambda^{2\tau }\right),$$

where $r_{0}=R^{2}\left(0\right)$ is the instantaneous symbol-decoding quality and $r_{0}\lambda^{2\tau }$ is the baseline memory attributable to input autocorrelation alone, derived in Appendix A.3. At ε=0.25 (λ=0) this baseline vanishes and $C_{\mathrm{mem}}^{\mathrm{corr}}$ reduces to the raw capacity.

#### 2.4.3. Mutual Information

We estimated the joint mutual information $I(S_{k-\tau };\;X_{k})$ between the discrete lagged symbol $S_{k-\tau }$ and the continuous *K*-dimensional reservoir state $X_{k}$ with the *k*-nearest-neighbor estimator of Ross [18], denoted $\hat{I}$. The estimator is purpose-built for mixed discrete–continuous pairs: for each sample, it selects a local radius from the *k*-th nearest neighbor within the same class, counts all cross-class samples within that radius, and applies digamma corrections to obtain an asymptotically unbiased estimate without binning the continuous variable. We used k=5 neighbors and the Chebyshev ($L_{\infty }$) metric. Estimates are normalized by the marginal entropy H(S)=2bits to give ${\mathrm{MI}}_{\mathrm{norm}}=\hat{I}/H\left(S\right)\in \left[0,1\right]$.

## 3. Results

We organize the findings in two parts: a spontaneous characterization that fixes the network size and validates the regime structure of the Brunel phase diagram, followed by an input-driven characterization that quantifies separation, memory, the role of input coupling density, the dependence on input statistics, and model-free validation using mutual information.

### 3.1. Spontaneous Characterization

**Figure A1:**
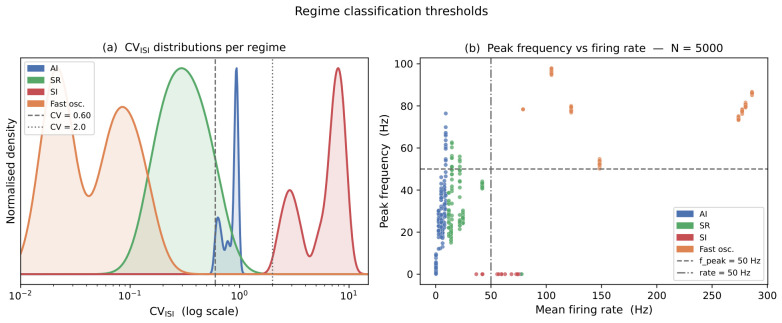
Empirical basis for spontaneous regime classification thresholds. (**a**) normalized kernel-density estimates of the mean ${\mathrm{CV}}_{\mathrm{ISI}}$ for each active regime, pooled across all network sizes (N=100–10,000) and all (g,η) conditions assigned to that regime. Densities are estimated on a log-transformed scale (bandwidth 0.25) and normalized to unit peak. Dashed and dotted vertical lines mark the classification thresholds at ${\mathrm{CV}}_{\mathrm{ISI}}=0.60$ (SR | AI boundary) and ${\mathrm{CV}}_{\mathrm{ISI}}=2.0$ (AI | SI boundary), respectively. The SR and AI distributions overlap near the boundary; the threshold at 0.60 is placed between their peaks (≈0.42 and ≈0.90). The SI distribution is well-separated above ${\mathrm{CV}}_{\mathrm{ISI}}=2.0$. (**b**) Peak spectral frequency $f_{\mathrm{peak}}$ versus mean population firing rate for all (g,η) conditions at N=5000. The fast-oscillation regime occupies the upper-right quadrant defined by $f_{\mathrm{peak}}>50\;\mathrm{Hz}$ and rate >50Hz (dashed and dash-dotted lines); all other active regimes remain below both thresholds.

**Figure A2:**
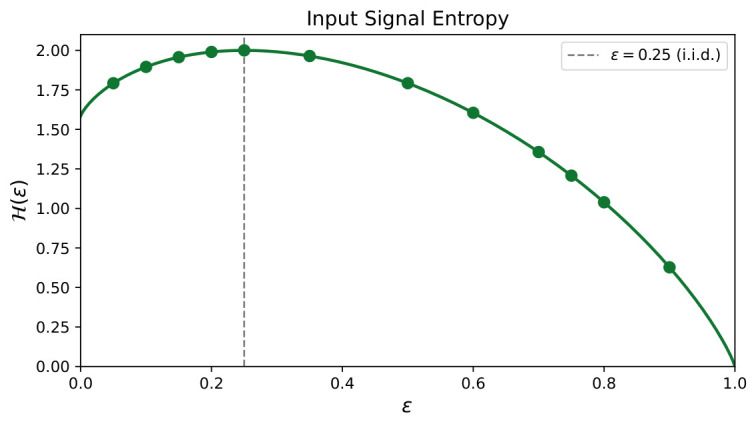
Entropy rate H(ε) (Equation (3)) as a function of the stay probability ε for $N_{\mathrm{in}}=4$ (solid curve). The dashed vertical line marks ε=0.25 (i.i.d., maximum entropy); filled circles indicate the ε values used in the driven experiments.

We first verified that the analytically predicted Brunel phase structure is reproduced in our finite-size simulations and identified a working size at which all four regimes coexist. We swept the (g,η) grid at seven network sizes (N∈{100,…,10,000}) and classified each condition into one of five regimes (quiescent, AI, SR, SI, fast oscillations) using empirical thresholds on mean firing rate, ${\mathrm{CV}}_{\mathrm{ISI}}$, $f_{\mathrm{peak}}$, and $\tau_{\mathrm{net}}$ (Figure A1, Appendix A.1).

#### 3.1.1. Phase Diagram

**Figure 1:**
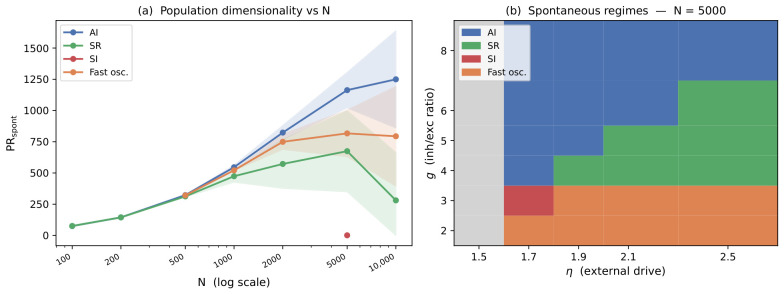
Spontaneous regime characterization. (**a**) Population dimensionality (${\mathrm{PR}}_{\mathrm{spont}}$) as a function of network size *N*, shown separately for each active regime (mean ± std over all (g,η) conditions assigned to that regime and all seeds). The AI regime scales approximately linearly with *N*; the SI regime collapses to ${\mathrm{PR}}_{\mathrm{spont}}\approx 1$ at large *N*, reflecting a rank-1 population structure dominated by a single slow oscillatory mode. (**b**) Modal spontaneous regime in the (η,g) parameter plane at N=5000 (modal classification over 10 seeds per condition). Color code: blue = AI, green = SR, red = SI, orange = fast oscillation; quiescent conditions (η=1.5) are omitted. The diagram reproduces the phase structure predicted by Brunel [5].

The phase diagram at N=5000 (Figure 1b) matches the theoretical prediction of Brunel [5]: η=1.5 is uniformly quiescent, low inhibitory ratios (g≤3) produce collective oscillations (SI at low drive, fast oscillations at higher η), and intermediate to high values of *g* produce SR or AI activity, with the SR–AI boundary shifting toward higher η as *g* increases. Per-regime statistics (Table 2) confirm the regime identities: AI shows low population synchrony (χ≈0.001, where χ is the Brunel population synchrony index defined as the ratio of the variance of the mean population activity to the mean single-neuron variance), moderate firing rate (ν≈4Hz), and irregular spiking (${\mathrm{CV}}_{\mathrm{ISI}}\approx 0.82$); SR shows regular spiking (${\mathrm{CV}}_{\mathrm{ISI}}\approx 0.39$) at higher rate (ν≈22Hz); SI shows extremely slow dynamics ($\tau_{\mathrm{net}}\approx 14\;s$); and fast oscillations show near-periodic firing at ∼80Hz. The network timescale $\tau_{\mathrm{net}}$ spans nearly three orders of magnitude across regimes, from ∼50 ms in fast oscillations to ∼14 s in SI. Two deviations from the original Brunel nomenclature arise from our fast fixed synapses ($\tau_{\mathrm{syn}}=0.5\;\mathrm{ms}$): the asynchronous regular (AR) regime is not accessible because it requires slow or distributed synaptic time constants, and Brunel’s unified SI regime splits into two empirically distinguishable branches (slow-oscillation SI and fast oscillations) that merge only when the synaptic time constant is long enough to prevent separation of their spectral peaks (see Appendix A.1 for full classification criteria).

#### 3.1.2. Population Dimensionality

Beyond regime classification, we quantified the effective dimensionality of spontaneous population activity, which is the substrate on which input-driven computation builds. The participation ratio ${\mathrm{PR}}_{\mathrm{spont}}$ (Figure 1a) reveals a sharp dissociation between regimes. In the AI regime, ${\mathrm{PR}}_{\mathrm{spont}}$ scales approximately linearly with *N*, rising from ∼75 at N=100 to ∼1250 at N=10,000: each additional neuron contributes a nearly independent degree of freedom, a hallmark of genuinely high-dimensional desynchronized activity. SI collapses to ${\mathrm{PR}}_{\mathrm{spont}}\approx 1$ at N=5000, with population covariance dominated by a single slow oscillatory mode regardless of size; SR and fast oscillations occupy intermediate, *N*-dependent positions. Spontaneous dimensionality is therefore regime-determined and size-dependent, and only the AI regime offers a high-dimensional state space available as a substrate for input-driven computation.

### 3.2. Input-Driven Characterization

We then quantified the computational capabilities of each regime by driving the N=5000 network with the four-state Markov input (Section 2.2). All experiments used the calibrated operating point $n_{\mathrm{frac}}=0.10$, $j_{\mathrm{in}}=J_{E}$, K=20 PCA components, and $T_{\mathrm{bit}}=50\;\mathrm{ms}$ (calibration sweeps in Appendix A.5); the main phase-diagram sweep fixed ε=0.25 (i.i.d., λ=0), removing input autocorrelation as a confound and isolating genuine reservoir-generated memory. Ten independent seeds were run per (g,η) condition.

#### 3.2.1. Separation Capacity

We computed kernel quality (KQ) (Equation (4)) across the full (g,η) phase plane under i.i.d. input (ε=0.25), where the null baseline is KQ=1 (equal between- and within-symbol distances). All conditions exceed this null value (Table 3): even in the fast-oscillation regime (g=2), states driven by different symbols are 3.1–3.8 times farther apart than states driven by the same symbol. KQ rises systematically with *g* and depends only weakly on η: at g=5 (canonical AI) KQ reaches 4.8–5.3, and at g=8 it reaches 5.4–5.8. To verify that this trend does not plateau within the swept range, we extended the sweep to g∈{10,12,16} at η=2.1; KQ continues to increase (5.73±0.09, 5.79±0.09, 5.85±0.09, respectively) with diminishing increments, consistent with saturation at higher *g* but without a clear plateau within the explored range. The systematic dependence on *g* is consistent with the progressive decorrelation of single-neuron responses with increasing inhibitory balance, which expands the volume of population state space available for separating distinct inputs and tracks the spontaneous dimensionality results.

#### 3.2.2. Transient Memory Capacity

To test whether the same inhibitory-balance gradient that governs separation also determines memory, we computed the corrected linear memory capacity $C_{\mathrm{mem}}^{\mathrm{corr}}$ (Equation (6)) across the same (g,η) grid under i.i.d. input. Because the memory profile is dominated by a single lag (see below), $C_{\mathrm{mem}}^{\mathrm{corr}}\approx R^{2}\left(1\right)$, ranging from 0 (no recoverable memory) to 1 (perfect recovery of the previous symbol’s variance). The phase diagram (Table 4) shows a strong dependence on *g* and a weak dependence on η at fixed *g*: memory is essentially zero for g≤3 (SI and fast-oscillation conditions), marginal at g=4 ($C_{\mathrm{mem}}^{\mathrm{corr}}\leq 0.18$), and rises sharply for g≥5, reaching 0.611 at g=8 (η=2.1). An extension to g∈{10,12,16} at η=2.1 confirms the monotonic trend (0.621±0.006, 0.630±0.006, 0.642±0.005) with diminishing increments; no plateau is observed within the explored range. The strongest memory therefore remains in the strongly inhibited region (g≥6), and the upper bound is not yet reached at g=16.

At N=10,000, the AI representative (g=5, η=1.9) yields $C_{\mathrm{mem}}^{\mathrm{corr}}=0.553\pm 0.006$, confirming the result is not a finite-size artifact of N=5000; the full regime characterization is conducted at N=5000 where the phase structure is stable across all four dynamical states (Appendix A.2).

**Figure 2:**
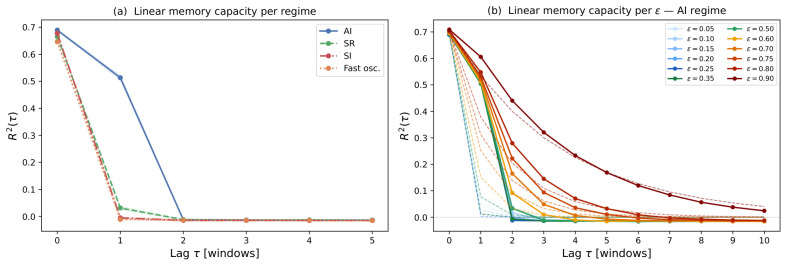
Memory lag profiles. (**a**) Cross-validated $R^{2}\left(\tau \right)$ as a function of lag τ (in symbol windows) for each regime representative at ε=0.25, $T_{\mathrm{bit}}=50\;\mathrm{ms}$ (mean ± std over 10 seeds). All regimes show a predominantly single-lag structure; the AI regime has the highest $R^{2}\left(1\right)$. (**b**) $R^{2}\left(\tau \right)$ for the AI regime across the full ε grid. Solid lines show the raw lag curves; dashed lines of the same color show the input autocorrelation baseline $r_{0}\lambda^{2\tau }$. At ε=0.25 (λ=0) the baseline vanishes; at higher ε it absorbs an increasing fraction of the raw $R^{2}$.

The lag-by-lag profile (Figure 2a) shows a predominantly single-lag structure across all regimes: $R^{2}\left(1\right)$ accounts for nearly all of $C_{\mathrm{mem}}^{\mathrm{corr}}$, while $R^{2}\left(\tau \right)$ falls to the noise floor for τ≥2. The dependence of this profile on the symbol window duration relative to the spontaneous network timescale is examined in Appendix A.9.

#### 3.2.3. Dead-Reservoir Baseline

To confirm that the transient memory reported above is a genuine product of the recurrent spiking dynamics rather than an artifact of the sparse input projection, we compared the intact reservoir against a *dead-reservoir* control: an identical network, with the same parameters and input wiring as the canonical AI condition (g=5, η=1.9, $n_{\mathrm{frac}}=0.10$, ε=0.25), but with all recurrent connections (E→E, E→I, I→E, and I→I) removed, so that only the feed-forward external noise and the four input projections drive the neurons. Table 5 shows the different metrics in the two conditions.

Corrected memory collapses to zero without recurrence, confirming that the memory measured throughout the paper is carried by recurrently sustained population activity and cannot arise from the instantaneous input projection alone. Separation, by contrast, does not depend on recurrence: KQ is in fact marginally higher in the dead reservoir, because instantaneous class separability is already provided by the channel-specific sparse input projection, and because the absence of correlated recurrent fluctuations slightly sharpens the within-class scatter. This double dissociation, in which memory is abolished but separation is preserved, isolates precisely which capability requires the recurrent spiking dynamics and provides a sharper control than a generic linear or rate-based baseline: it identifies recurrence as the specific source of transient memory while showing that separation has a purely feed-forward origin.

#### 3.2.4. Population Dimensionality Under Input

To uncover the underlying basis of the separation and memory results, we compared population dimensionality in the spontaneous and input-driven states for the four canonical regime representatives (Table 6). Input drive reduces PR in every regime, but the regime ordering reverses: ${\mathrm{PR}}_{\mathrm{spont}}$ is highest in AI (∼930) and lowest in SI (∼1), whereas ${\mathrm{PR}}_{\mathrm{driven}}$ is lowest in AI (∼4.6) and highest in fast oscillations (∼15.6). The inversion reflects a qualitative change in what dimensionality indicates: under drive, low PR means that input-evoked responses concentrate along a small number of highly informative directions rather than spread uniformly. This concentration enables compact, well-separated class representations and, in the AI regime, supports both high KQ and high $C_{\mathrm{mem}}^{\mathrm{corr}}$ from the same geometric mechanism.

#### 3.2.5. Input Coupling Density and Timescale Collapse

The phase-diagram results above were obtained under sparse input coupling ($n_{\mathrm{frac}}=0.10$). To establish whether this design choice is incidental or essential to the regime ordering, we swept $n_{\mathrm{frac}}\in \left\{0.01,0.02,0.05,0.10,0.25,0.50\right\}$ at the canonical AI operating point and recorded the driven mean firing rate, the driven network timescale, and $C_{\mathrm{mem}}^{\mathrm{corr}}$ (Table 7; full sweeps in Appendix A.7, with calibration of $j_{\mathrm{in}}$ and *K* in Appendix A.8 and Appendix A.6). Memory capacity peaks at $n_{\mathrm{frac}}=0.10$ ($C_{\mathrm{mem}}^{\mathrm{corr}}=0.513\pm 0.009$) and decreases monotonically as coupling becomes either sparser or denser: it falls sharply to 0.051±0.007 at $n_{\mathrm{frac}}=0.50$ and more gradually to 0.380±0.012 at $n_{\mathrm{frac}}=0.01$.

The two regimes of sub-optimal coupling are mechanistically distinct. At high $n_{\mathrm{frac}}$, the driven mean rate rises from ∼8 Hz to ∼110 Hz and the driven network timescale collapses to ∼3 ms; consecutive symbol windows become dynamically independent, and memory drops to near zero (0.051±0.007 at $n_{\mathrm{frac}}=0.50$). The collapse is structural, not a tuning artifact: no value of $j_{\mathrm{in}}$ or $T_{\mathrm{bit}}$ recovers memory at $n_{\mathrm{frac}}=0.50$ (Appendix A.8 and Appendix A.9), and dense coupling eliminates memory across all four regimes, not only in AI. At low $n_{\mathrm{frac}}$, by contrast, the network timescale remains short (close to the spontaneous value of ∼6 ms) and no dynamical collapse occurs, but the sparse input perturbation leaves a weaker signal in the population state; capacity falls gradually from 0.513±0.009 at $n_{\mathrm{frac}}=0.10$ to 0.380±0.012 at $n_{\mathrm{frac}}=0.01$, a reduction consistent with progressively weaker perturbation of the recurrent state. Sparse input coupling is therefore the operating regime in which the network’s intrinsic dynamics, and the regime differences they induce, become measurable; without it, the inhibitory-balance ordering of memory documented above cannot be observed in any spiking architecture of this class.

#### 3.2.6. Dependence on Input Entropy Rate

**Figure 3:**
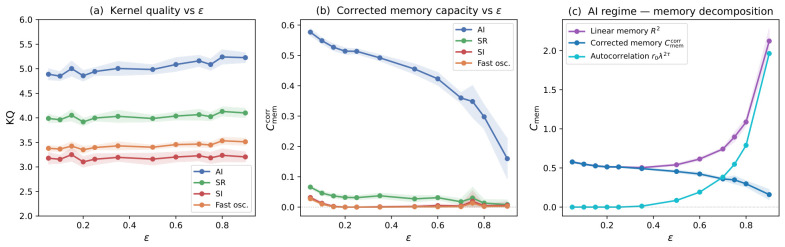
Dependence of separation and memory on input entropy rate, parameterized by the stay probability ε (H(ε) given by Equation (3)). (**a**) Kernel quality (KQ) as a function of ε for each regime representative (mean ± std over 10 seeds). KQ is flat across the full range, confirming that separation capacity is approximately invariant under changes in input entropy rate. (**b**) Corrected memory capacity $C_{\mathrm{mem}}^{\mathrm{corr}}$ vs. ε. All regimes show a monotone decrease as entropy falls; the AI regime dominates at every entropy level. (**c**) Decomposition of raw linear memory capacity (purple) into the genuine reservoir memory component (dark blue) and the baseline input autocorrelation contribution (cyan). At maximum entropy (ε=0.25, λ=0) the baseline vanishes; at low entropy (ε=0.90, λ=0.867) it absorbs most of the raw capacity. Fixed: $T_{\mathrm{bit}}=50\;\mathrm{ms}$, $n_{\mathrm{frac}}=0.10$, K=20.

A separate question is whether the regime differences in KQ and $C_{\mathrm{mem}}^{\mathrm{corr}}$ are properties of the network alone or are co-determined by the information content of the input. We swept the source entropy rate H(ε) over its full range, from i.i.d. (ε=0.25, H=2bits/symbol, λ=0) to strongly redundant (ε=0.90, H≈0.69bits/symbol, λ≈0.87), at the four regime representatives and with all network parameters held fixed (Figure 3). KQ is flat across the entire range (variation below 8%, Figure 3a). KQ depends on the reservoir-state scatter, not on the symbol-state scatter, and reservoir-state correlations with past symbols propagate only through genuine memory; because corrected memory is small relative to the marginal variance in every regime, the within-class scatter inherits a near-uniform marginal, leaving KQ approximately ε-invariant. The residual variation under 8% is consistent with this account, and separation is therefore an approximately ε-invariant, purely geometric property of the recurrent dynamics [10,14]. $C_{\mathrm{mem}}^{\mathrm{corr}}$, in contrast, decreases monotonically as H falls (Figure 3b): the decomposition (Figure 3c) shows that, at low entropy, the slow input baseline $r_{0}\lambda^{2\tau }$ absorbs most of the raw $R^{2}\left(\tau \right)$, while at maximum entropy (ε=0.25, λ=0) this baseline vanishes and the full network-added memory is exposed. The AI regime dominates $C_{\mathrm{mem}}^{\mathrm{corr}}$ at every entropy level tested, and the regime ordering is preserved throughout, so the AI advantage is structural rather than an artifact of the i.i.d. baseline.

#### 3.2.7. Mutual Information with Past Inputs

**Figure 4:**
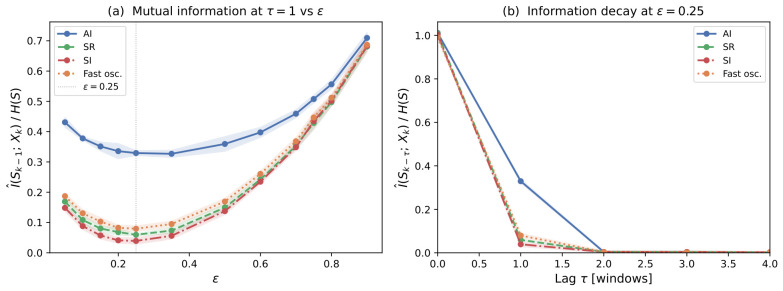
Non-parametric mutual information between past input symbols and reservoir state, estimated using the Ross (2014) [18] k-NN estimator (k=5) applied to the 20-dimensional PCA state. Results are normalized by the marginal input entropy H(S)=2bits. (**a**) Normalized MI at lag τ=1 as a function of ε for each regime. MI increases with ε for all regimes due to growing input autocorrelation, not increased reservoir memory; the AI advantage is maintained throughout. (**b**) Information decay curve: normalized MI vs. lag τ at ε=0.25 (i.i.d. input). The AI regime encodes 33% of H(S) at τ=1, dropping to the noise floor at τ≥2. All other regimes encode ≤8% at τ=1.

For a model-free validation of the linear memory results, we estimated the joint mutual information $I(S_{k-\tau };\;X_{k})$ between past input symbols and the current 20-dimensional PCA reservoir state with the Ross k-NN estimator (Section 2.4.3), and normalized it by H(S)=2bits to obtain a fractional encoding measure in [0,1]. At ε=0.25 and τ=1, AI encodes 33% of H(S), compared with 8% for fast oscillations, 6% for SR, and 4% for SI (Figure 4b), exactly the regime ordering of $C_{\mathrm{mem}}^{\mathrm{corr}}$. The information decay confirms the single-lag profile: I/H(S) drops from 0.33 at τ=1 to 0.004 at τ=2 and reaches the noise floor for τ≥3. As ε increases (entropy rate decreases, redundancy grows), ${\mathrm{MI}}_{\mathrm{norm}}$ rises in every regime (Figure 4a), but the rise is fully attributable to the growing temporal redundancy of the source rather than to enhanced reservoir integration, and the AI advantage holds throughout. The absolute normalized MI values depend on the estimator parameter *k*: in 20-dimensional PCA state space with ∼2000 samples, the Ross estimator produces larger estimates at small *k* and smaller ones at large *k*, spanning roughly a nine-fold range across k∈{3,5,10,20} (Appendix A.10). The regime ordering AI > fast oscillations > SR > SI is, however, preserved across all tested values of *k*, confirming that the qualitative conclusion is not a consequence of the specific choice k=5.

## 4. Discussion

We mapped the regime-to-computation relationship by traversing the Brunel phase diagram and quantifying separation (KQ) and transient memory ($C_{\mathrm{mem}}^{\mathrm{corr}}$) at every operating point under a Markov input source whose entropy rate could be tuned at fixed marginal distribution. Both capabilities rise monotonically with the inhibitory ratio *g* and remain jointly highest in the strongly inhibited asynchronous irregular region, a pattern that admits a coherent mechanistic reading. Stronger inhibition enforces asynchronous irregular firing [4,5] and decorrelates single-neuron responses through tracking between excitatory and inhibitory currents [28], expanding the dimensionality of the population state (visible in the near-linear scaling of ${\mathrm{PR}}_{\mathrm{spont}}$ with *N*, and in the gap between regimes at the canonical representative points: ${\mathrm{PR}}_{\mathrm{spont}}\approx 933$ in AI vs. ≈1.2 in SI, Table 6). The expanded state space supports separation, because more independent directions are available to embed distinct inputs [29]; the same decorrelation supports memory, because different input histories leave distinguishable traces instead of collapsing onto shared modes. Both capabilities thus increase together rather than trading off as in classical formulations [10,17], and we frame *g* as a unified architectural control parameter. This interpretation is consistent with rate-network evidence that adaptation toward balance improves reservoir performance [30] and with theoretical work linking cortical operating points to inhibitory balance [31], but it is established here within a homogeneous random architecture and should be tested in structured or plastic networks before being transferred without modification.

The synchronous irregular regime offers the sharpest test of whether $\tau_{\mathrm{net}}$ alone can predict memory. A timescale-matching argument [14] would place SI at the top of the ranking on the strength of its ∼14 s spontaneous timescale; the opposite holds, with $C_{\mathrm{mem}}^{\mathrm{corr}}\approx 0$ throughout. The dimensionality measurement resolves the paradox: with ${\mathrm{PR}}_{\mathrm{spont}}\approx 1$, SI activity is locked onto a single slow mode, so distinct symbols project onto nearly indistinguishable population states. A long timescale is necessary but not sufficient; the population must also carry enough intrinsic dimensionality for input traces to occupy separable directions. We note that $C_{\mathrm{mem}}^{\mathrm{corr}}$ captures only a narrow slice of temporal computation, namely, the linear recovery of the previous symbol; the total (linear and nonlinear) information processing capacity of any reservoir is bounded by *N* [17], and our value of ∼0.51 occupies only a small fraction of this budget, leaving open whether nonlinear capabilities follow the same regime ordering.

That the AI regime supports memory at all is, in turn, surprising in the opposite direction: its spontaneous timescale (∼6 ms) is much shorter than the symbol duration ($T_{\mathrm{bit}}=50\;\mathrm{ms}$), and short-timescale networks of this kind are not obvious candidates for behaviorally relevant temporal tasks. The advantage rests on population geometry rather than timescale: the high-dimensional state space (${\mathrm{PR}}_{\mathrm{spont}}\propto N$) provides many independent directions in which short-lived transients, sampled at the right moment, can carry substantial information. The actionable design target is therefore dimensionality, raised by increasing *g*, rather than $\tau_{\mathrm{net}}$, which the fast alpha-synapse architecture constrains intrinsically. Behaviorally relevant integration windows can be obtained either by an external low-pass readout or by augmenting the model with slow synaptic components, both of which decouple input integration from the recurrent connectivity that supplies dimensionality and separation. We emphasize that the memory measured here is the fading, history-into-state trace of reservoir computing [9], distinct from the attractor-based working memory studied in Brunel–Wang networks [32,33]: such slow components would extend the former in our architecture rather than produce the latter, which requires bistability and structured feedback not present here.

The absolute KQ values reported here (3.1–5.9) lie well below those of Legenstein and Maass [14] with comparable metrics, but a direct numerical comparison is not meaningful: we evaluate KQ in a K=20 PCA-compressed state space rather than the full $N_{E}$-dimensional state, and our input is deliberately perturbative (each channel targets only 10% of neurons through standard-weight synapses, $j_{\mathrm{in}}=J_{E}$). The within-class denominator is therefore dominated by spontaneous fluctuations rather than input-evoked variance. The perturbative regime is not a deficiency but the very condition under which memory survives [23,34] and the regime in which formal linear-response approaches to spiking networks are valid [35]; KQ should be read against the null value of one. That all regimes nonetheless reach 3–6 times the null while the input represents only a small fraction of the total excitatory drive is, in our view, the more informative comparison.

The behavior of memory under variations of input coupling density (Appendix A.7) makes input sparsity a structural design parameter rather than a calibration choice. At $n_{\mathrm{frac}}=0.50$ the driven mean rate rises to ∼110 Hz and the driven network timescale collapses to ∼3 ms; consecutive symbol windows become dynamically independent, and only residual memory survives (0.051±0.007, roughly 10% of the maximum). The onset lies between $n_{\mathrm{frac}}=0.25$ and $n_{\mathrm{frac}}=0.50$, and the collapse is robust to weight and symbol duration (Appendix A.8), ruling out compensation through tuning. Mechanistically, the input ceases to perturb the recurrent network and instead overrides it: when half the neurons are externally driven at fixed rates, recurrent self-organization no longer determines population activity. The driven-period power spectrum clarifies the nature of this collapse (Supplementary Material, Section S3 and Figure S1): the spectrum stays broadband and flat at every coupling density, with no peak emerging at high $n_{\mathrm{frac}}$, which rules out a synchronization transition and instead indicates rate saturation, an increasing share of the population being locked to the memoryless, input-driven current rather than to the recurrently sustained activity that carries memory. This is the spiking-network counterpart of established results in rate networks where input correlations and stimulus-driven chaos suppression reshape internal dynamics [23,24]. We characterized the transition by an empirical density sweep rather than by a closed-form linear-response or mean-field criterion because the collapse is a finite-size phenomenon: it depends on the number of directly driven neurons in a network of N=5000, whereas mean-field and linear-response treatments take the N→∞ limit, in which the driven fraction, not its absolute count, enters. The mean-field balance argument does predict the direction of the effect (Supplementary Material, Section S3), but the quantitative location of the transition at finite size is most directly obtained empirically. The operating point $n_{\mathrm{frac}}=0.10$ was calibrated in the AI regime; a regime-specific recalibration would tighten the comparison, although the absence of recovery in SI or fast oscillations under any weight multiplier suggests limited room for adjustment.

Taken together, these findings yield a two-level design principle for spiking reservoir computers: choose a high-*g* AI operating point to obtain the population dimensionality that both capabilities require, and keep input coupling sparse enough that the perturbation preserves the intrinsic dynamics rather than overriding them. The two choices are largely independent of each other and of the information content of the input source: separation is approximately invariant under changes in entropy rate, while the ranking of regimes by memory persists from i.i.d. to strongly redundant inputs. This information-theoretic robustness, made possible by the closed-form control that the symmetric Markov construction affords over the entropy rate at fixed marginal, provides a quantitative reference against which extensions to structured connectivity, heterogeneous neurons, or plasticity [36,37,38] can be evaluated. Read the other way, the same principle restates a familiar machine-learning heuristic, control representational dimensionality and avoid overdriving the recurrent state, in the language of inhibitory balance and population geometry.

Several limitations bound the scope of the conclusions. The computational assessment relies on linear readout metrics (kernel quality and linear memory capacity, corroborated by mutual information); the optimality of the AI regime is therefore established for linear separation and memory, and whether it extends to strongly nonlinear temporal tasks remains to be tested. The model is the homogeneous random Brunel network with exclusively fast alpha-function synapses; no slow excitatory component, spike-frequency adaptation, structured connectivity, or synaptic plasticity is included. These omissions are by design (to isolate the effect of inhibitory balance on a well-characterized phase diagram), but they preclude direct generalization to circuits where such components shape the operating regime: the Brunel mean-field balance condition is derived under the assumption $\tau_{\mathrm{syn}}\ll \tau_{\mathrm{mem}}$, and extensions to slow excitation require a complete reparametrization of the phase diagram rather than a substitution of $\tau_{\mathrm{syn}}$. Within the Brunel parametrization itself, the asynchronous regular regime of the original phase diagram is not accessible and has consequently not been tested. The readout uses the top K=20 PCA components of the excitatory population, and KQ is evaluated in this compressed space. We verified that the regime ordering is preserved under a full-state ridge readout and a random projection (Supplementary Material, Section S1), but absolute values should still be read as readout-dependent. The input alphabet was varied over $N_{\mathrm{in}}\in \left\{4,8,16\right\}$ symbols at the AI point (Supplementary Material, Section S2); separation is preserved while linear memory declines as the per-class sample budget shrinks, so the four-symbol results are representative of the separation behavior, but the memory values are specific to the alphabet size. Finally, the monotonic trend of KQ and $C_{\mathrm{mem}}^{\mathrm{corr}}$ with *g* does not plateau within the explored range (g≤16); whether a structural maximum exists at higher inhibitory ratios, and at what cost to mean firing rate and network stability, remains open.

Three directions follow naturally. First, augmenting the Brunel model with slow synaptic components (NMDA excitation, spike-frequency adaptation) would let us test whether the inhibitory-balance principle survives in networks that combine high dimensionality with behaviorally relevant timescales [32,33]. Second, testing whether the AI advantage transfers to nonlinear tasks (NARMA, temporal pattern classification) and to architectures with structured connectivity, heterogeneous neuron types, or activity-dependent plasticity would probe its generality beyond homogeneous random networks. Third, decomposing the mechanistic origin of the high-*g* advantage between single-neuron decorrelation and population-level geometry of input-evoked responses would convert the empirical regularity reported here into a predictive theory, with direct relevance for the design of neuromorphic hardware that targets cortical-style computation.

## 5. Conclusions

Our phase-wide characterization identifies inhibitory balance, indexed by *g*, as a primary determinant of reservoir computing performance in the Brunel network. Both separation and corrected memory rise monotonically with *g* across the full range tested, remaining jointly highest in the strongly inhibited asynchronous irregular region, while the synchronous irregular regime, despite a network timescale three orders of magnitude longer, supports neither capability. This constitutes a direct empirical counterexample to the network timescale as a sufficient predictor of memory: the timescale-matching hypothesis [14] would place the synchronous irregular regime at the top of the ranking on the strength of $\tau_{\mathrm{net}}\approx 14\;s$; the opposite holds, because the regime’s near-rank-one population geometry prevents distinct input histories from occupying separable directions regardless of how long the network’s collective mode persists. The decisive variable is therefore population dimensionality, set by inhibitory decorrelation [28,31], rather than slow relaxation alone. This refines a central design heuristic for biologically constrained reservoirs: a useful target is the operating point at which inhibitory balance maximally expands the state space, in line with recent evidence that locally adapting the E-I balance toward target firing rates substantially improves reservoir performance in rate networks [30].

A second distinctive contribution is the closed-form control of input entropy rate at fixed marginal that the symmetric Markov construction affords. Sweeping the source redundancy from i.i.d. to strongly correlated, with the symbol distribution held uniform throughout, reveals that separation is approximately invariant under such changes, while memory tracks the information content of the source monotonically through the baseline correction. Prior work on input-dependent memory in echo state networks [21] and autocorrelated sources [22] does not provide this axis of control, which is what allows us to attribute the regime ordering of memory unambiguously to network architecture rather than to input statistics.

A third contribution is the identification of input coupling density as a structural design parameter on equal footing with network architecture. Targeting 25% of neurons per channel already reduces memory capacity, and at 50% coupling, memory is effectively eliminated across all regimes through a collapse of the network timescale that no choice of synaptic weight or symbol duration can rescue. Sparse input projection is thus the condition under which regime differences become measurable in the first place, and should be reported and controlled in any benchmark of spiking reservoir performance.

More broadly, the analysis illustrates how a model from the computational-neuroscience tradition, evaluated with the metrics and information-theoretic instrumentation of reservoir computing, can ground RNN design heuristics in identifiable biophysical control parameters, and we expect this style of joint characterization to be most useful precisely where machine-learning methods are deployed on data of biological origin.

## Figures and Tables

**Table 1:** Reservoir network parameters. Values follow Brunel [5] unless noted.

Parameter	Symbol	Value
Total neurons	*N*	5000
Excitatory fraction	$N_{E}/N$	0.8
Inhibitory fraction	$N_{I}/N$	0.2
Connection probability	*c*	0.1
Membrane time constant	$\tau_{m}$	20 ms
Synaptic time constant	$\tau_{\mathrm{syn}}$	0.5 ms
Firing threshold	$V_{\mathrm{th}}$	20 mV
Reset potential	$V_{\mathrm{reset}}$	10 mV
Refractory period	$t_{\mathrm{ref}}$	2 ms
Excitatory weight	$J_{E}$	0.1 mV
Inhibitory ratio	*g*	{2,3,4,5,6,8} (full grid)
		{10,12,16} at η=2.1 (extension)
External drive	η	{1.5,1.7,1.9,2.1,2.5}

**Table 2:** Per-regime spontaneous activity statistics (N=5000, mean ± std over all seeds and (g,η) conditions classified as that regime). Quiescent conditions excluded. ^†^ The large std of $\tau_{\mathrm{net}}$ in AI and SR reflects a single boundary condition (g=3, η=1.7) with anomalously long timescale; interquartile ranges are [6.5,8.3] ms (AI) and [5.0,14.8] ms (SR). ^‡^ $f_{\mathrm{peak}}<0.1$ Hz in SI indicates the absence of a detectable oscillatory peak above the spectral floor, consistent with the ∼14 s autocorrelation timescale.

Metric	AI	SR	SI	Fast osc.
*n* (runs)	204	121	13	142
ν [Hz]	3.9±6.0	22.0±11.5	60.0±13.1	211±79
χ	0.001±0.001	0.006±0.002	0.021±0.013	0.001±0.001
${\mathrm{CV}}_{\mathrm{ISI}}$	0.82±0.12	0.39±0.11	6.19±2.48	0.04±0.02
$f_{\mathrm{peak}}$ [Hz] ^‡^	20±17	33±12	<0.1	78±12
$\tau_{\mathrm{net}}$ [ms] ^†^	14±93	16±54	14,459±9145	52±16
${\mathrm{PR}}_{\mathrm{spont}}$	1163±142	675±325	1.16±0.14	817±187

**Table 3:** Kernel quality KQ (mean ± std over 10 seeds) across the (g,η) phase plane. Spontaneous regime labels (modal over seeds at N=5000) shown in the rightmost column. Fixed: ε=0.25, $T_{\mathrm{bit}}=50\;\mathrm{ms}$, $n_{\mathrm{frac}}=0.10$, K=20.

*g*	η=1.7	η=1.9	η=2.1	η=2.5	Regime
2	3.61±0.06	3.79±0.06	3.64±0.04	3.09±0.03	fast_osc
3	3.24±0.12	3.59±0.09	3.79±0.07	3.95±0.08	SI/fast_osc
4	4.26±0.08	3.92±0.08	3.94±0.09	4.14±0.07	AI/SR
5	5.26±0.10	5.05±0.08	4.89±0.07	4.75±0.07	AI/SR
6	5.58±0.10	5.44±0.09	5.29±0.08	5.11±0.08	AI/SR
8	5.79±0.09	5.71±0.09	5.61±0.09	5.41±0.08	AI

**Table 4:** Corrected linear memory capacity $C_{\mathrm{mem}}^{\mathrm{corr}}$ (mean ± std over 10 seeds) across the (g,η) phase plane. Spontaneous regime labels (modal over seeds at N=5000) shown in the rightmost column. Fixed: ε=0.25, $T_{\mathrm{bit}}=50\;\mathrm{ms}$, $n_{\mathrm{frac}}=0.10$, K=20.

*g*	η=1.7	η=1.9	η=2.1	η=2.5	Regime
2	0.000±0.000	0.000±0.000	0.000±0.000	0.000±0.000	fast_osc
3	0.000±0.000	0.000±0.000	0.000±0.001	0.000±0.000	SI/fast_osc
4	0.180±0.011	0.091±0.005	0.061±0.011	0.031±0.007	AI/SR
5	0.434±0.012	0.513±0.009	0.448±0.005	0.284±0.011	AI/SR
6	0.400±0.011	0.537±0.009	0.587±0.005	0.503±0.006	AI/SR
8	0.407±0.010	0.538±0.007	0.611±0.005	0.606±0.005	AI

**Table 5:** Intact versus dead-reservoir (no recurrent connections) comparison at the canonical AI point (mean ± std over 10 seeds). Removing recurrence abolishes memory while leaving instantaneous separation intact.

	KQ	$C_{\mathrm{mem}}^{\mathrm{corr}}$	${\mathrm{PR}}_{\mathrm{driven}}$
Intact reservoir	5.05±0.08	0.513±0.009	4.56±0.02
Dead reservoir (no recurrence)	5.55±0.09	0.000±0.000	4.48±0.03

**Table 6:** Population dimensionality under spontaneous (${\mathrm{PR}}_{\mathrm{spont}}$) and input-driven (${\mathrm{PR}}_{\mathrm{driven}}$) conditions at N=5000, for the four canonical regime representative points. ${\mathrm{PR}}_{\mathrm{spont}}$: mean ± std over 20 seeds (SI: 13 seeds classified as SI at g=3, η=1.7; remaining 7 transition to other regimes). ${\mathrm{PR}}_{\mathrm{driven}}$, KQ: mean ± std over 10 seeds. Input driving reduces dimensionality in all regimes; the regime ordering reverses between the two conditions.

Regime	(g,η)	${\mathrm{PR}}_{\mathrm{spont}}$	${\mathrm{PR}}_{\mathrm{driven}}$	KQ
AI	(5,1.9)	933±13	4.56±0.02	5.05±0.08
SR	(4,2.5)	471±16	6.23±0.03	4.14±0.07
SI	(3,1.7)	1.16±0.14	6.08±0.09	3.24±0.12
Fast osc.	(2,1.9)	589±6	15.63±0.23	3.79±0.06

**Table 7:** Memory capacity, driven firing rate, and driven network timescale as a function of input coupling density $n_{\mathrm{frac}}$, AI regime (g=5,η=1.9; mean ± std over 10 seeds). Bold: selected operating point.

$n_{\mathrm{frac}}$	$\nu_{\mathrm{dr}}$ [Hz]	$\tau_{\mathrm{net},\mathrm{dr}}$ [ms]	$C_{\mathrm{mem}}^{\mathrm{corr}}$
0.50	109.64±0.76	2.70±0.15	0.051±0.007
0.25	45.10±0.36	2.03±0.10	0.419±0.008
**0.10**	18.94±0.25	3.06±0.07	0.513±0.009
0.05	12.42±0.21	3.90±0.07	0.487±0.010
0.02	8.85±0.16	5.45±0.10	0.448±0.014
0.01	7.63±0.16	6.49±0.13	0.380±0.012

## Data Availability

All the data used in this study can be generated using the scripts available at https://github.com/luigirosati/entropy-reservoir (accessed on 26 May 2026).

## Supplementary Materials

The following supporting information can be downloaded at: https://www.mdpi.com/article/10.3390/e28070784/s1, Figure S1: Power spectral density of driven population activity across input coupling densities; Table S1: Kernel quality and corrected memory at the four regime representatives under three readouts; Table S2: Kernel quality, corrected memory, and driven population dimensionality at the canonical AI point as a function of the input alphabet size.

## Abbreviations

The following abbreviations are used in this manuscript:

AI	Asynchronous irregular
CV	Coefficient of variation
E/I	Excitatory–inhibitory
i.i.d.	Independent and identically distributed
KQ	Kernel quality
LIF	Leaky integrate-and-fire
LSM	Liquid state machine
MI	Mutual information
PCA	Principal component analysis
PR	Participation ratio
RC	Reservoir computing
SI	Synchronous irregular
SNN	Spiking neural network
SR	Synchronous regular

## Appendix A

### Appendix A.1. Spontaneous Regimes Classification

Spontaneous activity was classified into different regimes using a sequential rule based on three metrics: mean population firing rate, coefficient of variation of the inter-spike interval (${\mathrm{CV}}_{\mathrm{ISI}}$), peak spectral frequency ($f_{\mathrm{peak}}$), and network autocorrelation timescale ($\tau_{\mathrm{net}}$). A condition was labeled quiescent if the mean rate fell at or below 0.1 Hz. Among active conditions, fast oscillation was identified by the joint criterion $f_{\mathrm{peak}}>50\;\mathrm{Hz}$ and mean rate >50Hz, which isolates the high-frequency synchronous regime in the lower-*g*, lower-η corner of the phase diagram (Figure A1b). The remaining conditions were classified using ${\mathrm{CV}}_{\mathrm{ISI}}$: synchronous irregular (SI) required both ${\mathrm{CV}}_{\mathrm{ISI}}>2.0$ and $\tau_{\mathrm{net}}>1000\;\mathrm{ms}$, consistent with the slow, burst-dominated dynamics described by Brunel [5]; synchronous regular (SR) was assigned when ${\mathrm{CV}}_{\mathrm{ISI}}<0.60$; all remaining conditions were labeled asynchronous irregular (AI). The thresholds were set empirically by inspecting the ${\mathrm{CV}}_{\mathrm{ISI}}$ distributions pooled across all network sizes and parameter combinations (Figure A1a).

This classification uses five active states rather than Brunel’s original four: the AR (asynchronous regular) regime is not accessible with our fast fixed synapses, while Brunel’s SI regime is split into slow-oscillation SI and fast-oscillation branches because the short synaptic time constant prevents them from merging.

### Appendix A.2. Network Size Selection

The spontaneous characterization sweep covered seven network sizes (N∈{100,200,500,1000,2000,5000,10,000}) with 10 seeds per (g,η) condition. The AI and SR regimes are present at all *N*: asynchronous and synchronous regular dynamics arise from single-neuron properties and do not require large-scale synchrony. The SI and fast-oscillation regimes, by contrast, depend on collective synchrony and are suppressed by finite-size fluctuations at small *N*. Fast oscillations first appear reliably above N≈2000; the SI regime is absent below N≈5000, where noise prevents the slow burst cycles from sustaining coherently across the population. At N≤1000, conditions that would be SI or fast oscillation at large *N* are mis-classified as SR, because the collective behavior has not yet emerged. The network size N=5000 is therefore the smallest at which all four regimes can be observed simultaneously, making it the natural operating point for regime-comparative experiments.

### Appendix A.3. Markov Source Autocorrelation

The symmetric $N_{\mathrm{in}}$-state Markov chain defined by Equation (2) has a circulant transition matrix. Its eigenvalues are 1 (multiplicity 1, corresponding to the uniform stationary distribution) and

(A1) $$\lambda =\frac{N_{\mathrm{in}}\varepsilon -1}{N_{\mathrm{in}}-1},$$

with multiplicity $N_{\mathrm{in}}-1$. For $N_{\mathrm{in}}=4$ this gives λ=(4ε−1)/3. Because the non-trivial eigenvalues are degenerate, the lag-τ autocorrelation of any zero-mean function of the chain equals $\lambda^{\tau }$ exactly. For $\varepsilon >1/N_{\mathrm{in}}$ (λ>0) the autocorrelation decays monotonically, with input timescale $\tau_{\varepsilon }=-T_{\mathrm{bit}}/\ln\lambda$; for $\varepsilon <1/N_{\mathrm{in}}$ (λ<0) the autocorrelation alternates in sign and $\tau_{\varepsilon }$ is undefined; at $\varepsilon =1/N_{\mathrm{in}}$ exactly, λ=0 and the sequence is i.i.d. The baseline term $r_{0}\lambda^{2\tau }$ subtracted in Equation (6) follows directly: it is the expected lag-τ$R^{2}$ under a readout that tracks the input autocorrelation alone, without any genuine reservoir memory.

### Appendix A.4. Simulation Parameters

Table A1 collects all simulation and readout parameters used in the input-driven experiments. The calibrated values (*K*, $n_{\mathrm{frac}}$, $j_{\mathrm{in}}$, $T_{\mathrm{bit}}$) are justified by the sweeps in Appendix A.6, Appendix A.7, Appendix A.8 and Appendix A.9; all other values are fixed by the experimental design.

**Table A1:** Simulation and readout parameters for the input-driven experiments. Calibrated values are marked with (⋆); fixed design choices are unmarked.

Parameter	Symbol	Value	Note
Symbol windows	$N_{w}$	2000	per condition
Symbol duration	$T_{\mathrm{bit}}$	50 ms	(⋆) Appendix A.9
Input channels	$N_{\mathrm{in}}$	4	
Stay probability	ε	0.25 (main); 0.05–0.90 (sweep)	
Targeting fraction	$n_{\mathrm{frac}}$	0.10	(⋆) Appendix A.7
Input weight	$j_{\mathrm{in}}$	$J_{E}$	(⋆) Appendix A.8
ON rate	$\nu_{\mathrm{ON}}$	100 Hz	
Warm-up duration	$T_{\mathrm{warm}}$	2 s	
Spontaneous recording	$T_{\mathrm{spont}}$	120 s	
PCA components	*K*	20	(⋆) Appendix A.6
Random seeds	—	10 per (g,η)	vary connectivity + targets

### Appendix A.5. Input Coupling and Readout Parameter Calibration

The input-driven experiments depend on three design choices (the number of PCA components used for the readout (*K*), the fraction of neurons targeted by the input ($n_{\mathrm{frac}}$), and the input synaptic weight ($j_{\mathrm{in}}$)) none of which has a principled closed-form optimum. We determined these parameters empirically by sweeping each one while fixing all others, using the corrected linear memory capacity $C_{\mathrm{mem}}$ in the AI regime (g=5, η=1.9) as the target metric. The symbol window duration $T_{\mathrm{bit}}$ was additionally characterized to situate our operating point relative to the timescale-matching hypothesis. The results of all four sweeps are reported below.

### Appendix A.6. PCA Readout Dimension

Linear memory capacity is computed from the principal components of the driven population spike-count matrix. The number of retained components *K* determines the dimensionality of the readout state and directly affects the estimated capacity: too few components discard the dimensions that carry the memory signal, whereas too many add noise dimensions that inflate the variance explained by chance.

We performed a sweep on K∈{5,10,20,40,80} at the canonical AI operating point (g=5, η=1.9, $n_{\mathrm{frac}}=0.10$, $j_{\mathrm{in}}=J_{E}$, $T_{\mathrm{bit}}=50\;\mathrm{ms}$, ε=0.25). The results are shown in Table A2.

**Table A2:** Corrected linear memory capacity $C_{\mathrm{mem}}$ (mean ± std over 10 seeds) as a function of the number of PCA components *K*, AI regime (g=5, η=1.9). Bold: selected operating point.

**K**	5	10	**20**	40	80
$C_{\mathrm{mem}}$	0.001±0.002	0.382±0.007	0.513±0.009	0.520±0.006	0.513±0.007

The memory signal is essentially invisible at K=5 and recovers progressively as *K* is increased, saturating between K=20 and K=40. This behavior indicates that the information about past inputs is distributed across dimensions 6–20 of the PCA spectrum, not in the dominant modes of the population response. Above K=40, capacity is flat or slightly decreasing as noise components dilute the estimate. We adopt K=20 as the operating point: it captures the full memory signal at roughly half the computational cost of K=40, and the 1.4% difference in $C_{\mathrm{mem}}$ between the two is smaller than the across-seed variability.

### Appendix A.7. Input Targeting Fraction

This appendix supports the input-coupling result reported in Section 3 (Table 7). Each of the four input channels drives a dedicated subset of excitatory and inhibitory neurons, with $n_{\mathrm{frac}}$ controlling the fraction of $N_{E}$ (and $N_{I}$) targeted per channel. We swept $n_{\mathrm{frac}}\in \left\{0.01,0.02,0.05,0.10,0.25,0.50\right\}$ at fixed K=20, $j_{\mathrm{in}}=J_{E}$, $T_{\mathrm{bit}}=50\;\mathrm{ms}$, ε=0.25, and the canonical AI operating point. The per-condition results (Table 7 in the main text) document the timescale collapse at $n_{\mathrm{frac}}=0.50$: the mean excitatory rate rises to ∼110 Hz, $\tau_{\mathrm{net}}$ drops to ∼3 ms, and $C_{\mathrm{mem}}^{\mathrm{corr}}$ falls to near zero (0.051). The collapse is robust to weight scaling (it persists even at $j_{\mathrm{in}}=0.1\;J_{E}$, not shown), confirming that it is the number of driven neurons rather than per-synapse strength that overrides the recurrent dynamics. At $n_{\mathrm{frac}}=0.25$ the rate rises to ∼45 Hz but $C_{\mathrm{mem}}^{\mathrm{corr}}$ remains substantial (0.419), indicating that the onset of the collapse lies between $n_{\mathrm{frac}}=0.25$ and $n_{\mathrm{frac}}=0.50$. At small $n_{\mathrm{frac}}$ (≤0.05), the driven timescale remains close to the spontaneous value and no collapse occurs, but the weaker input signal reduces readout signal-to-noise; capacity falls gradually to 0.380 at $n_{\mathrm{frac}}=0.01$. We adopt $n_{\mathrm{frac}}=0.10$ as the operating point that maximizes $C_{\mathrm{mem}}^{\mathrm{corr}}$ while keeping coupling perturbative.

### Appendix A.8. Input Synaptic Weight

The parameter $j_{\mathrm{in}}$ sets the strength of each input synapse as a multiple of the standard excitatory weight $J_{E}$. We performed a sweep on $j_{\mathrm{in}}\in \left\{0.3,0.5,1.0,2.0,3.0\right\}\times J_{E}$ at fixed $n_{\mathrm{frac}}=0.10$ in all four regime representatives (Table A3).

**Table A3:** Corrected linear memory capacity $C_{\mathrm{mem}}$ (mean ± std over 5 seeds) as a function of the input weight multiplier $j_{\mathrm{in}}/J_{E}$, for each regime ($n_{\mathrm{frac}}=0.10$, $T_{\mathrm{bit}}=50\;\mathrm{ms}$, ε=0.25). **Bold**: selected operating point.

$j_{\mathrm{in}}/J_{E}$	AI	SR	SI	Fast osc.
0.3	0.305±0.016	0.001±0.002	0.000±0.000	0.000±0.000
0.5	0.464±0.010	0.004±0.004	0.000±0.000	0.000±0.000
**1.0**	0.516±0.006	0.035±0.006	0.000±0.000	0.000±0.000
2.0	0.451±0.012	0.040±0.011	0.000±0.000	0.000±0.000
3.0	0.450±0.010	0.034±0.011	0.000±0.000	0.000±0.000

In the AI regime, $C_{\mathrm{mem}}$ peaks at $j_{\mathrm{in}}=J_{E}$ and is approximately flat above $j_{\mathrm{in}}=1.0\;J_{E}$, while at $j_{\mathrm{in}}=0.3\;J_{E}$ it is considerably reduced. Below $j_{\mathrm{in}}\approx 0.5\;J_{E}$, the input perturbation is too weak to reliably separate symbol representations in the population state; above $j_{\mathrm{in}}=1.0\;J_{E}$, stronger synapses begin to suppress recurrent dynamics and capacity plateaus or slightly decreases. No weight multiplier unlocks memory in the SI or fast-oscillation regimes, confirming that the capacity advantage of AI is structural rather than a tuning artefact. We adopt $j_{\mathrm{in}}=J_{E}$ as a principled, parameter-free choice: it matches the standard recurrent weight and sits at the optimum without requiring regime-specific tuning.

### Appendix A.9. Symbol Window Duration and the Timescale-Matching Hypothesis

The symbol window duration $T_{\mathrm{bit}}$ sets the temporal resolution of the input and determines how much time the reservoir has to integrate information within each symbol before the readout is taken. As discussed in Section 2.2, choosing $T_{\mathrm{bit}}$ is inherent to any rate-to-spike encoding scheme: it is the integration window over which a downstream rate readout would average the spike train, and any such protocol must commit to a value. A naive timescale-matching argument would predict that memory capacity peaks when $T_{\mathrm{bit}}\approx \tau_{\mathrm{net}}$: if $T_{\mathrm{bit}}\ll \tau_{\mathrm{net}}$ the network has not relaxed between symbols and the readout conflates adjacent symbols; if $T_{\mathrm{bit}}\gg \tau_{\mathrm{net}}$ the transient evoked by each symbol decays completely before the readout, so no memory trace reaches the next window.

We performed a sweep on $T_{\mathrm{bit}}\in \left\{10,20,50,100\right\}\;\mathrm{ms}$ in the AI regime at ε=0.25 (Table A4). The spontaneous network timescale at this operating point is $\tau_{\mathrm{net},\mathrm{sp}}\approx 6\;\mathrm{ms}$.

**Table A4:** Corrected linear memory capacity $C_{\mathrm{mem}}$ (mean ± std over 10 seeds) as a function of symbol window duration $T_{\mathrm{bit}}$, AI regime (g=5, η=1.9, $n_{\mathrm{frac}}=0.10$, ε=0.25).

$T_{\mathrm{bit}}$ **[ms]**	10	20	50	100
$C_{\mathrm{mem}}$	0.768±0.027	0.626±0.008	0.513±0.009	0.503±0.037

Capacity decreases monotonically with $T_{\mathrm{bit}}$ over the range tested, with no bell-curve peak visible. This is consistent with the timescale-matching hypothesis: our standard operating point of $T_{\mathrm{bit}}=50\;\mathrm{ms}$ is approximately 8× the spontaneous network timescale, well into the regime where each window is dynamically near-independent. The predicted optimum lies below $T_{\mathrm{bit}}=10\;\mathrm{ms}$, but very short windows are practically constrained by synaptic delay artifacts (minimum axonal delay ∼1ms, creating cross-window bleed at $T_{\mathrm{bit}}≲5\;\mathrm{ms}$).

The apparent mismatch between the model timescale ($\tau_{\mathrm{net},\mathrm{sp}}\approx 6\;\mathrm{ms}$) and the biologically motivated $T_{\mathrm{bit}}=50\;\mathrm{ms}$ is primarily a consequence of the synaptic model used in the Brunel network rather than an argument against the chosen input timescale. The Brunel model employs exclusively fast alpha-function synapses ($\tau_{\mathrm{syn}}=0.5\;\mathrm{ms}$) with no slow excitatory component (NMDA receptors, τ∼50–150ms) and no slow inhibition (GABA_B_, τ∼100–200ms). In real cortical circuits, these slow synaptic components substantially extend the effective network timescale; empirical autocorrelation times of population activity range from ∼20ms in primary sensory areas to ∼300ms in prefrontal cortex. By contrast, $T_{\mathrm{bit}}=50\;\mathrm{ms}$ is directly motivated by the timescales of behaviorally relevant signals that cortical circuits process: phonemes in continuous speech (50–150 ms), beta-band oscillation cycles (33–77 ms at 13–30 Hz), sensory integration windows, and motor command intervals all fall in this range. Processing inputs at $T_{\mathrm{bit}}\approx \tau_{\mathrm{net},\mathrm{sp}}\approx 6\;\mathrm{ms}$ would require stimuli changing at ∼170Hz, well above the rates at which most cortical computations operate. We therefore retain $T_{\mathrm{bit}}=50\;\mathrm{ms}$ as the biologically grounded operating point, and interpret the observed monotone decrease in capacity as motivation for extending the Brunel model with slow synaptic dynamics, a natural direction for future work.

### Appendix A.10. MI Estimator Robustness (k-NN Parameter)

The Ross (2014) [18] k-nearest-neighbor estimator is applied to the 20-dimensional PCA reservoir state with ∼2000 sample windows per condition. In high dimensions with finite samples, the absolute MI estimate depends on *k*: larger *k* increases the effective neighborhood radius and yields smaller MI estimates (conservative bias), while smaller *k* yields larger estimates (liberal bias). To quantify this sensitivity, we re-ran the estimator for k∈{3,5,10,20} on the four canonical regime representatives at ε=0.25 (Table A5).

The absolute normalized MI at lag τ=1 spans roughly a nine-fold range across the tested *k* values, as expected from the known finite-sample properties of the estimator. The regime ordering AI > fast oscillation > SR > SI is, however, preserved at every value of *k*, confirming that the qualitative conclusion is not an artefact of the specific choice k=5 used in the main analysis.

**Table A5:** Non-parametric mutual information $I_{\mathrm{Ross}}\left(S_{k-1};\;X_{k}\right)/H\left(S\right)$ at lag τ=1 for the four canonical regime representatives (N=5000, ε=0.25), as a function of the *k*-NN estimator parameter. Mean ± std over 10 seeds. The absolute values decrease with *k* as expected for the Ross (2014) [18] estimator in 20-dimensional state space with finite sample size; the regime ordering AI > fast oscillations. > SR > SI is preserved across all tested *k*.

Regime	k=3	k=5	k=10	k=20
AI	0.439±0.007	0.329±0.008	0.166±0.011	0.048±0.007
SR	0.093±0.008	0.060±0.004	0.026±0.006	0.011±0.005
SI	0.065±0.009	0.039±0.011	0.015±0.005	0.006±0.003
Fast osc.	0.111±0.009	0.080±0.010	0.037±0.012	0.011±0.009
