# Supplementary material for: Entropy, Inhibition and Memory in Balanced Spiking Reservoirs
Source: Entropy (Basel). 2026 Jul 10;28(7):784. doi: 10.3390/e28070784 (PMC13409623; doi:10.3390/e28070784)
Supplement: Supplementary file 1 [file entropy-28-00784-s001.zip › entropy-4370156-supplementary.pdf]

Supplementary Materials

1. Readout-Independence of the Regime Ordering

The main-text analyses use a K-dimensional principal component analysis (PCA) projection of the excitatory population spike-count matrix ($K = 20$ for memory and mutual information, $K = 20$ for kernel quality; see Appendix A of the main text for the dimension sweep). To confirm that the regime ordering does not depend on this choice of readout, we recomputed kernel quality (KQ) and corrected linear memory capacity ($C_{\mathrm{mem}}^{\mathrm{corr}}$) at the four canonical regime representatives (AI: $g = 5$, $\eta = 1.9$; SR: $g = 4$, $\eta = 2.5$; SI: $g = 3$, $\eta = 1.7$; fast oscillations: $g = 2$, $\eta = 1.9$; all at $\varepsilon = 0.25$, $T_{\mathrm{bit}} = 50\,\mathrm{ms}$) using three readouts:

1. **PCA** (as in the main text): the $K = 20$ leading principal components of the excitatory population state.
2. **Full state:** the complete $N_E = 4{,}000$-dimensional excitatory population state, with ridge-regularized, cross-validated linear regression for $C_{\mathrm{mem}}^{\mathrm{corr}}$ (ordinary least squares is ill-posed when the number of features exceeds the number of samples).
3. **Random projection:** a fixed $d = 100$ random projection of the full state, with ordinary least-squares $C_{\mathrm{mem}}^{\mathrm{corr}}$.

Regime membership was verified independently per seed from dedicated 120-second spontaneous recordings (same connectivity seed as the driven run), using the classification rule of the main-text spontaneous characterization. This matters at the SI point, where only 7 of 10 seeds are robustly classified as SI; the remaining 3 transition to a neighboring regime and were excluded.

Table S1. Kernel quality (KQ) and corrected memory ($C_{\mathrm{mem}}^{\mathrm{corr}}$) at the four regime representatives under three readouts (mean ± std). The AI regime ranks first for separation and is the only regime with substantial memory under every readout. Absolute values shrink under the full state and random projection because PCA concentrates the discriminative subspace and raises the signal-to-noise ratio, but the leading structure, which is the claim made in the main text, is unaffected.

	KQ		$C_{\mathrm{mem}}^{\mathrm{corr}}$		
Regime	PCA	Full	PCA	Ridge (full)	Rand. proj.
AI	5.05 ± 0.08	3.68 ± 0.05	0.513 ± 0.009	0.298 ± 0.016	0.184 ± 0.010
SR	4.14 ± 0.07	2.62 ± 0.03	0.031 ± 0.007	0.000 ± 0.000	0.000 ± 0.000
SI	3.21 ± 0.10	2.35 ± 0.05	0.000 ± 0.000	0.000 ± 0.000	0.000 ± 0.000
Fast osc.	3.79 ± 0.06	1.67 ± 0.01	0.000 ± 0.000	0.000 ± 0.000	0.000 ± 0.000

On both the PCA and the full state, the AI regime ranks first for separation, and the leading gap AI > SR is preserved. Among the two weakest regimes the order of SI and fast oscillations depends on the readout (fast oscillations rank above SI under PCA, below it on the full state), but this reordering concerns only the bottom of the ranking and does not affect the central claim, which concerns the AI regime. Corrected memory is substantial only in the AI regime on all three readouts; the small PCA value for SR (0.031) vanishes under both the ridge-regularized full-state and the random-projection readouts, so AI is the only regime carrying robust memory. The absolute values are smaller under the full state and the random projection, an expected consequence of PCA denoising the discriminative subspace, but the identification of AI as the separation-and-memory optimum, which is the claim of the paper, is independent of the choice of readout.

2. Dependence on Input Alphabet Size

The main text uses a four-symbol Markov source. To test whether the results generalize beyond this vocabulary, we repeated the key measurements at the canonical AI point ($g = 5$, $\eta = 1.9$, $\varepsilon = 0.25$, $T_{\mathrm{bit}} = 50\,\mathrm{ms}$) with $N_{\mathrm{in}} \in \{4, 8, 16\}$ input symbols, 10 seeds each.

Table S2. Kernel quality, corrected memory, and driven population dimensionality at the canonical AI point as a function of the input alphabet size N_{in} (mean ± std over 10 seeds).

N_{in}	KQ	$C_{\mathrm{mem}}^{\mathrm{corr}}$	$\mathrm{PR}_{\mathrm{driven}}$
4	5.05 ± 0.08	0.513 ± 0.009	4.56 ± 0.02
8	4.77 ± 0.12	0.472 ± 0.016	9.55 ± 0.06
16	4.89 ± 0.15	0.114 ± 0.007	19.02 ± 0.16

Separation (KQ) is essentially unchanged as the alphabet grows from 4 to 16 symbols: the reservoir continues to map each symbol to a well-separated state. Population dimensionality ($\mathrm{PR}_{\mathrm{driven}}$) scales almost exactly linearly with N_{in}, showing that the reservoir allocates an additional effective mode per extra symbol rather than saturating. Corrected memory decreases at $N_{\mathrm{in}} = 16$; this is a readout capacity-budget effect rather than a failure of the recurrent dynamics. With 16 symbols, each state occupies only ∼125 of the 2,000 driven windows, so the $K = 20$-dimensional linear readout must estimate 15 additional one-hot target columns from substantially fewer examples per class. The reservoir's ability to separate inputs (KQ) is unaffected; only the linear decodability of the past symbol degrades as the per-class sample budget shrinks.

3. Mechanism of the Timescale Collapse under Dense Coupling

The main text reports that dense input coupling ($n_{\mathrm{frac}} = 0.50$) collapses the driven network timescale and abolishes memory. To identify the mechanism, we computed the population-activity power spectral density (PSD) during the driven period at six coupling densities, $n_{\mathrm{frac}} \in \{0.01, 0.02, 0.05, 0.10, 0.25, 0.50\}$, at the same AI condition, 10 seeds each (Figure S1).

The spectra are broadband and essentially flat at every n_{frac}: there is no sharp spectral peak at any coupling density, and in particular no peak emerges at high n_{frac}. The only systematic effect of increasing n_{frac} is a vertical shift of the whole spectrum (more driven neurons produce proportionally more power at all frequencies), not a change in spectral shape. A weak, broad excess near ∼20 Hz is visible at low n_{frac}, consistent with the network's intrinsic timescale-related fluctuation; it does not sharpen or grow with coupling density.

This rules out a synchronization account: the network does not develop a collective rhythm under dense coupling. The data instead support a *rate-saturation* account. As n_{frac} grows, a larger fraction of neurons receive strong direct, non-recurrent drive at each active window. Because g and η are unchanged, the structural inhibition-to-excitation ratio is untouched, but the *effective* drive each targeted neuron receives is the sum of its recurrent input and a large, input-locked current that does not depend on the symbol history. As $n_{\mathrm{frac}} \to 0.5$, an increasing share of the population response is dominated by this instantaneous, memoryless drive rather than by the recurrently sustained activity that carries memory. The population code is diluted, not desynchronized. This is the mechanistic counterpart of the memory collapse reported in the main text, and it is a rate-saturation rather than a synchronization transition.

Consistent with this account, the Brunel mean-field balance condition is stated for recurrently generated input; when a fraction n_{frac} of neurons is additionally driven by a fixed external current independent of network state, the balance argument no longer governs the driven subpopulation, whose firing is set by the external drive rather than by

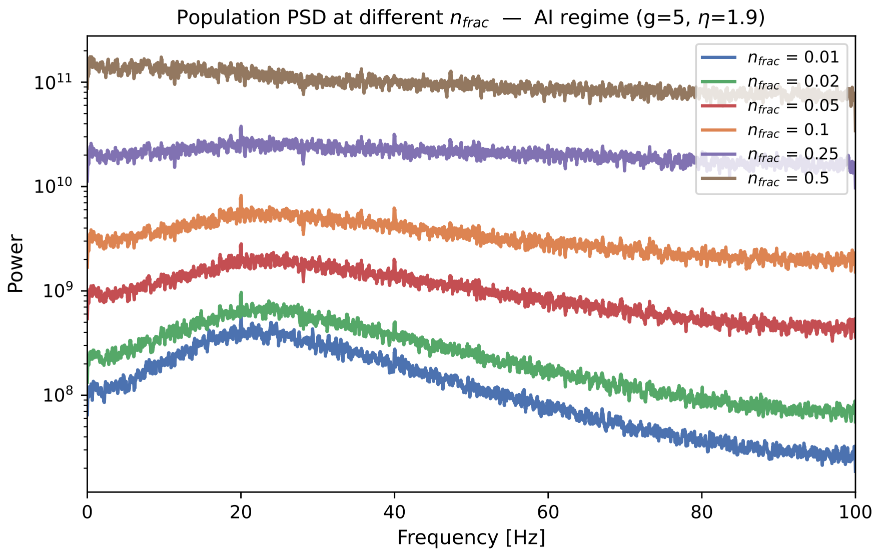

Figure S1. Power spectral density of driven population activity across input coupling densities. Power spectral density (PSD) of the excitatory population spike-count signal (binned at 5 ms, averaged over 10 network seeds) during the driven period, computed at six values of the input coupling density $n_{\mathrm{frac}} \in \{0.01, 0.02, 0.05, 0.10, 0.25, 0.50\}$. All other parameters correspond to the canonical AI condition ($g = 5$, $\eta = 1.9$, $\varepsilon = 0.25$, $T_{\mathrm{bit}} = 50\,\mathrm{ms}$). Spectra are shown on a logarithmic power axis and smoothed with a 15-point moving average for visual clarity. Increasing n_{frac} raises the overall power level but does not produce or sharpen any spectral peak: the spectra remain flat across the entire frequency range, ruling out a transition to synchronous oscillation. A weak broad excess near 20 Hz, visible at low n_{frac}, is consistent with the network's intrinsic timescale τ_{net}; it neither grows nor sharpens as coupling density increases. The absence of synchrony supports a rate-saturation interpretation of the memory collapse observed at high n_{frac} (main text, Section 3.2).

the excitatory-inhibitory cancellation that sustains asynchronous irregular activity. The transition is therefore expected to depend primarily on the number of driven neurons, consistent with its robustness to per-synapse weight reported in Appendix A8 of the main text.
